# Corticotropin Releasing Factor in the Bed Nucleus of the Stria Terminalis in Socially Defeated and Non-stressed Mice with a History of Chronic Alcohol Intake

**DOI:** 10.3389/fphar.2017.00762

**Published:** 2017-10-25

**Authors:** Lucas Albrechet-Souza, Thiago W. Viola, Rodrigo Grassi-Oliveira, Klaus A. Miczek, Rosa M. M. de Almeida

**Affiliations:** ^1^Institute of Psychology, Federal University of Rio Grande do Sul, Porto Alegre, Brazil; ^2^Developmental Cognitive Neuroscience Lab (DCNL) and Brain Institute (InsCer), Pontifical Catholic University of Rio Grande do Sul, Porto Alegre, Brazil; ^3^Departments of Psychology and Neuroscience, Tufts University, Medford, MA, United States

**Keywords:** alcohol, elevated plus-maze, tail suspension test, anxiety, extended amygdala, BNST, CRF, CRF receptors

## Abstract

Stress exposure has been identified as one risk factor for alcohol abuse that may facilitate the transition from social or regulated use to the development of alcohol dependence. Preclinical studies have shown that dysregulation of the corticotropin releasing factor (CRF) neurotransmission has been implicated in stress-related psychopathologies such as depression and anxiety, and may affect alcohol consumption. The bed nucleus of the stria terminalis (BNST) contains CRF-producing neurons which seem to be sensitive to stress. In this study, adult male C57BL/6 mice previously defeated in resident-intruder confrontations were evaluated in the elevated plus-maze and tail suspension test. Mice were also tested for sweet solution intake before and after social stress. After having had continuous access to ethanol (20% weight/volume) for 4 weeks, control and stressed mice had CRF type 1 (CRFR1) or type 2 (CRFR2) receptor antagonists infused into the BNST and then had access to ethanol for 24 h. In separate cohorts of control and stressed mice, we assessed mRNA levels of BNST *CRF, CRFR1* and *CRFR2*. Stressed mice increased their intake of sweet solution after ten sessions of social defeat and showed reduced activity in the open arms of the elevated plus-maze. When tested for ethanol consumption, stressed mice persistently drank significantly more than controls during the 4 weeks of access. Also, social stress induced higher BNST *CRF* mRNA levels. The selective blockade of BNST CRFR1 with CP376,395 effectively reduced alcohol drinking in non-stressed mice, whereas the selective CRFR2 antagonist astressin2B produced a dose-dependent increase in ethanol consumption in both non-stressed controls and stressed mice. The 10-day episodic defeat stress used here elicited anxiety- but not depressive-like behaviors, and promoted an increase in ethanol drinking. CRF-CRFR1 signaling in the BNST seems to underlie ethanol intake in non-stressed mice, whereas CRFR2 modulates alcohol consumption in both socially defeated and non-stressed mice with a history of chronic intake.

## Introduction

Alcoholics often refer to stress and anxiety as strong motivators for drinking (Ludwig and Wikler, 1974; Litman et al., 1983; Sinha, 2009). In fact, ethanol is well established as stress-relieving in both laboratory animals and humans, and the tension reduction hypothesis remains one of the oldest theories proposed to explain why individuals consume ethanol (Conger, 1956). Several experimental methods have been developed to increase voluntary ethanol drinking in laboratory animals, but it has been challenging to reliably and adequately characterize the stress-alcohol relationship (Becker et al., 2011; Noori et al., 2014).

Social defeat and subordination stress can lead to increased ethanol drinking in mice and monkeys compared with non-stressed animals or higher-ranking individuals (Peretti and Lewis, 1969; Sillaber et al., 2002; McKenzie-Quirk and Miczek, 2008). In addition to increased ethanol consumption (Norman et al., 2015), repeated episodes of social defeat stress have been demonstrated to promote an enhancement of dopamine release in the mesolimbic pathway in response to a stimulant challenge (Han et al., 2015). Furthermore, 10 days of episodic social defeat stress induced a chronic elevation in plasma corticosterone in outbred mice, indicating altered hypothalamic-pituitary-adrenal (HPA) stress function (Norman et al., 2015). This cascade of neuroendocrine responses is initiated by corticotropin releasing factor (CRF), which integrates adaptive physiological responses to stress (Owens and Nemeroff, 1991).

The primary role of CRF is to activate the HPA axis by increasing the release of glucocorticoids (Vale et al., 1981). In parallel, CRF axons project to extrahypothalamic areas, mediating neurovegetative and behavioral responses to stress (Vale et al., 1983) that underlie vigilance, fear, and emotionality (Merlo Pich et al., 1995; Heinrichs and Koob, 2004; Schulkin et al., 2005). These projection areas include the amygdala, bed nucleus of the stria terminalis (BNST) and ventral tegmental area (VTA) (Swanson et al., 1983; Sawchenko et al., 1993). The CRF system in mammals is composed of the CRF and three other CRF-like peptides, including urocortin (Ucn) 1, Ucn2, and Ucn3 (Hauger et al., 2003; Bale and Vale, 2004). The effects of CRF and Ucns are mediated by two receptors, namely CRF type 1 (CRFR1) and CRF type 2 (CRFR2), and a CRF binding protein (Hauger et al., 2003; Bale and Vale, 2004). The ligands present differences in the binding profile to CRF receptors. For instance, CRF has 10-fold higher affinity for CRFR1 than for CRFR2, while Ucn2 and Ucn3 bind with 100-fold higher affinities to the CRFR2 (Hauger et al., 2003).

The CRF system is critical for survival, but chronic overactivity can lead to stress-related pathologies, including anxiety, depression and alcohol abuse (Hundt et al., 2001; Gold and Chrousos, 2002; Southwick et al., 2005; de Kloet et al., 2008). Studies in laboratory animals, including lower mammals and primates, have shown that an up-regulation of the CRF system can underlie anxiety- and depression-like phenotypes (Kalin et al., 2000; Strome et al., 2002; Servatius et al., 2005; Jaferi and Bhatnagar, 2007), and lead to excessive alcohol drinking (Nie et al., 2004; Breese et al., 2005; Funk C.K. et al., 2006; Hansson et al., 2006; Sommer et al., 2008). Besides, acute drug withdrawal increases CRF activity in the amygdala, promoting a negative emotional state that motivates resumption and maintenance of drug taking (Funk C.K. et al., 2006; Roberto et al., 2010).

The extended amygdala, particularly the BNST, has been proposed as a critical site of action for adaptations associated with alcohol abuse (Koob, 2008; Silberman and Winder, 2013), and pharmacological manipulations in the BNST can alter alcohol drinking behaviors (Hyytiä and Koob, 1995; Eiler et al., 2003). Moreover, chronic alcohol exposure and withdrawal alter the function and plasticity of BNST neurons (Kash et al., 2009; McElligott and Winder, 2009). The BNST is also involved in behavioral responding to sustained fear through control of brain regions that mediate specific aspects of anxiety-like behavior (Walker and Davis, 2008).

The heterogeneous nature of the BNST, subdivided into at least 16 subregions and distinct cell types, creates two opposing circuits involved in the modulation of anxiety. Stress can differentially affect these circuitries within the BNST to shift the balance from an anxiolytic to an anxiogenic state (Jennings et al., 2013; Kim et al., 2013; Daniel and Rainnie, 2016; Henckens et al., 2016). For example, the anterior and posterior sections of the BNST serve opposing roles in the mediation of the HPA axis, respectively implicated in its activation and inhibition (Boudaba et al., 1996; Choi et al., 2007). Moreover, The BNST contains CRF-producing neurons which seem to be sensitive to stress (Cummings et al., 1983; Dabrowska et al., 2013). In fact, exposures to corticosterone and the pharmacological stressor yohimbine upregulate CRF mRNA expression in the BNST (Makino et al., 1994; Funk D. et al., 2006).

Although both CRF receptors are expressed within the BNST (Van Pett et al., 2000; Dabrowska et al., 2011; Rinker et al., 2017), few experimental studies have directly examined the role of BNST CRF in the interaction between stress and alcohol consumption, and little is known about the specific contribution of CRFR2. The current experiments were designed to test the hypothesis that brief episodes of social defeat stress can elicit dysregulated behaviors in adult C57BL/6 mice, including anxiety- or depressive-like symptoms and excessive ethanol consumption, as well as neuroadaptations of the CRF system in the BNST. Further, we investigated the effects of BNST treatment with either selective CRFR1 or CRFR2 antagonists on free-choice home cage ethanol drinking in non-stressed controls and stressed mice with a history of continuous access to ethanol.

## Materials and Methods

### Mice and Housing

Mice were bred at Federal University of Pelotas (Pelotas, RS, Brazil) and transported to the Animal Experimentation Unit from the Hospital de Clínicas de Porto Alegre (Porto Alegre, RS, Brazil). Upon arrival male C57BL/6 mice were 6 weeks of age and weighed 20–25 g and male and female Swiss mice were 6 weeks of age and weighed 25–30 g. They were housed in polycarbonate cages (30 × 18 × 15 cm) with pine shavings and allowed to habituate to the environment for 2 weeks before experimental procedures were initiated. Each male Swiss mouse (*n* = 12) was pair-housed with a ligated female Swiss mouse (*n* = 12), whereas C57BL/6 mice were housed individually (*n* = 85) in a separate room. Sterilized rodent laboratory chow (Nuvilab CR1; Quimtia, Colombo, PR, Brazil) and sterilized water were available *ad libitum* through stainless steel wire mesh lids. Swiss mice were maintained on a 12-h light/dark cycle (lights on 0700 h, lights off 1900 h), with constant temperature (22 ± 2°C) and humidity (50–60%). C57BL/6 mice were maintained on a 12-h partially reversed light/dark cycle (lights on 0300 h, lights off 1500 h), with constant temperature (22 ± 2°C) and humidity (50–60%). This study was carried out in accordance with the Brazilian Federal Law N°11.794/2008 for the scientific use of animals. The protocol was approved by the Ethics Committee on Animal Use of Animal Experimentation Unit from Hospital de Clínicas de Porto Alegre.

### Tubal-Ligation Surgery

Female Swiss mice were tubally ligated using antiseptic techniques and standard surgical procedure (Remie, 2000). Briefly, mice were anesthetized with ketamine (100 mg/kg) + xylazine (10 mg/kg, i.p.) and placed in the right lateral decubitus position, a dorsal incision (approximately 1.0 cm) was made, the ovary was located and the end of the uterine horn was tied off using absorbable sutures. The oviduct was located and severed using a micro-scissor. All reproductive structures were repositioned back in the abdominal cavity, and the abdominal incision was closed with absorbable sutures and the skin with non-absorbable sutures (Harris and Saltzman, 2013). The same procedure was performed on the left side. Mice were injected with tramadol (10 mg/kg, i.p.) immediately after the surgery and during the next three consecutive days (12/12 h) to provide analgesia. Female mice were single-housed and allowed to recover for 7 days before being paired with Swiss male mice. Upon termination of the experiment, females were euthanized with an overdose of ketamine (300 mg/kg) + xylazine (30 mg/kg, i.p.).

### Social Defeat Stress

After 3 weeks of pair-housing with a female, each male Swiss mouse was individually assessed for aggression in confrontations with male C57BL/6 mice assigned as “instigators” (*n* = 12). In the absence of the female cagemate, the number of attack bites by the Swiss mouse was recorded for 5 min. This procedure was performed for 5 consecutive days. Swiss mice that were determined to be reliably aggressive (more than 15 bites in 5 min) were used as “residents.” After the screening for resident’s aggressive behavior, instigators were euthanized with an overdose of ketamine (300 mg/kg) + xylazine (30 mg/kg, i.p.).

**FIGURE 1:**
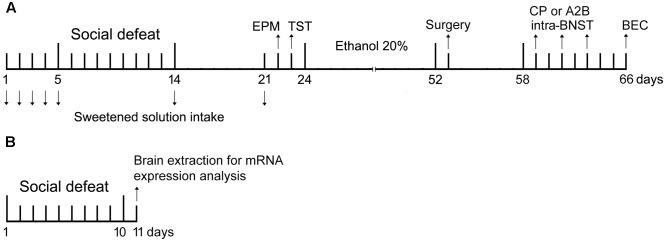
Experiment design. **(A)** Timeline refers to behavioral analysis and blood ethanol concentration (BEC) in non-stressed controls and socially defeated mice. On day 1, mice were exposed to sweet solution two-bottle choice for 24 h (habituation). On days 2–4 (baseline intake), 5, 14, and 21 mice were offered sweet solution in the two-bottle choice procedure for 90 min. On days 5–14, stressed mice were weighed and socially defeated. Controls were weighed daily. On day 22, controls and socially defeated mice were tested in the elevated plus-maze (EPM), and next day in the tail suspension test (TST). On day 24, mice were given continuous access to ethanol and water for 4 weeks before the surgical procedure to implant cannulae into the bed nucleus of the stria terminalis (BNST). After recovery, mice were infused with selective CRF receptors antagonists (CP376,395 or astressin2B) and tested for alcohol drinking. Each mouse received three microinjections, with a 48-h interval between them, in a design that counterbalanced saline and drug treatments. On day 66, mice were euthanized and had the brains and blood samples collected for histological and BEC analysis. **(B)** Timeline refers to analysis of *CRF, CRFR1* and *CRFR2* mRNA expression in the BNST in non-stressed controls and socially defeated mice. On days 1–10, controls were weighed daily and stressed mice were weighed and socially defeated. On day 11, mice were euthanized and had the brains collected for mRNA expression analysis.

C57BL/6 mice in the non-stressed control group were weighed daily, while stressed mice (“intruders”) were weighed and then socially defeated for ten consecutive sessions (**Figure 1A**) using the following procedure, which consisted of the pre-defeat threat, defeat, and post-defeat threat phases (Yap and Miczek, 2007). This procedure was performed during the light phase of the light-dark cycle, between 0900 and 1200 h. The female cagemate was removed before the pre-defeat phase and kept in a holding cage until the end of the post-defeat threat phase.

In the pre-defeat threat phase, an intruder mouse was placed into a perforated acrylic tube (18 cm × 6 cm) and positioned into the home cage of a resident mouse for 5 min. Intruders faced a different resident during each confrontation to prevent habituation and diminished aggression. During the defeat phase, the intruder mouse was removed from the perforated tube and placed into the resident’s cage without protection. The defeat phase lasted until the intruder had received 5 bites from the aggressive resident. In the post-defeat threat phase, the intruder was placed back into the perforated acrylic tube in the resident’s cage for 10 min. Following the post-defeat threat phase, the intruder and the female were returned to their home cages.

### Sweet Solution Two-Bottle Choice

Five days before starting the social defeat stress, experimental mice were exposed to one 50-ml bottle containing a sweet solution (0.1% saccharin and sodium cyclamate in sterilized water; Zero-Cal, Hypermarcas S.A., São Paulo, SP, Brazil) and another 50-ml bottle containing sterilized water for 24 h in the home cage. We used saccharin and sodium cyclamate instead of sucrose because of the absence of caloric content, which may affect the rewarding properties (Lockie et al., 2015). During the next three consecutive days, mice were given a daily two-bottle free-choice home cage water and sweet solution drinking for 90 min, 1 h after the onset of the dark photoperiod (baseline intake). This procedure was repeated 5-6 h after the first and last social defeat, and again 7 days after the last confrontation (**Figure 1A**). To prevent side preference, the position of the bottles was switched between trials. Sweet solution and water consumption was measured by weighing the bottles. An empty “drip” cage served as a control for evaporation and spillage. Fluid loss in this control condition was deducted from individual intake values.

### Elevated Plus-Maze (EPM) Test

The basic EPM design was similar to that originally described by Lister (1987), with two open arms (30 cm × 5 cm) and two closed arms (30 cm × 5 cm × 15 cm) connected via a central platform (5 cm × 5 cm). The apparatus was constructed from wood and was raised to a height of 50 cm above the ground. All testing was conducted under dim illumination (one 60 W red light providing 45 lux at the open arm of the maze) during the dark phase of the light-dark cycle.

Eight days after the last social defeat (**Figure 1A**), non-stressed controls and stressed mice were transported to the experimental room during the last hour of the light phase and left undisturbed for at least 2 h prior to testing. They were placed individually in the center of the maze facing a closed arm and allowed 5 min of free exploration. Behavior was recorded with a video camera positioned above the maze. The apparatus was thoroughly cleaned after each test with 70% ethanol.

Behavioral analysis was performed manually by an observer blind to the conditions and consisted of percentage of open arms entries [(open/total) × 100], time spent in the open arms and frequency of closed arms entries (arm entry = all four paws into an arm).

### Tail Suspension Test (TST)

The TST is a mouse behavioral test useful in the screening of potential antidepressant drugs, and to assess manipulations that are expected to promote or affect depression-related symptoms such as behavioral despair (Steru et al., 1985). Nine days after the last social defeat (**Figure 1A**), during the light phase of the light-dark cycle, non-stressed controls and stressed mice were suspended on the edge of a shelf 75 cm above the ground by an adhesive tape placed approximately 1 cm from the tip of tail. The duration of immobility was manually recorded for 6 min. Mice are considered immobile when they hang passively and motionless (Vangeois et al., 1997).

### Ethanol Two-Bottle Free-Choice Paradigm

Ethanol (20% weight/volume) solutions were prepared in sterilized water from 92.8% ethyl alcohol (Zeppelin; Cachoeirinha, RS, Brazil). Ten days after the last confrontation, non-stressed controls and stressed mice were weighed daily and given continuous access to ethanol and sterilized water for 4 weeks (**Figure 1A**) as described by Hwa et al. (2011). The bottles (50 ml) were weighed daily and had the positions switched (left/right) to avoid side preference. Mice drank ethanol for 4 weeks, before surgical preparation for pharmacological treatment. During drug testing, fluid intakes were measured by assessing bottle weights before and 2-, 4-, and 24-h after drug manipulations, during the dark phase of the light-dark cycle. To control for evaporation or spillage, “drip” measurements (ca. 0.2 ml/24 h) were taken from bottles on an empty cage and subtracted from individual intakes.

### Stereotaxic Surgery and Microinjection Procedure

Mice were anesthetized with a combination of ketamine (100 mg/kg) + xylazine (10 mg/kg, i.p.) prior to surgery and were kept under isoflurane throughout the surgical procedure. Pre-surgical analgesia was induced with tramadol (10 mg/kg, i.p.). Mice were implanted with a dual-cannula system (Plastics One, Roanoke, VA, United States) to bilaterally target the BNST. The stereotaxic coordinates, according to Paxinos and Franklin (2001), were: +0.3 mm posterior to bregma, ±1.1 mm lateral to the midline, and 4.3 mm ventral to the dura (Pleil et al., 2015). After surgery, pain control was provided with tramadol (10 mg/kg, i.p.) during the next two consecutive days (12/12 h), and mice recovered for 5–6 days. Dummy cannulae and dust caps fitted the length of the cannulae while dual injectors protruded 0.1 mm past the cannulae.

On the day before the first test day, mice received 1 sham injection, consisting of insertion of the injectors into the cannulae for 3 min. On the test days, doses of the CRFR1 antagonist CP376,395 (0.25 and 0.5 μg/side, Bio-Techne; Minneapolis, MN, United States) or the CRFR2 antagonist astressin2B (0.25 and 0.5 μg/side, Tocris; Ellisville, MO, United States) were freshly dissolved in saline solution (NaCl 0.9%) and mice received bilateral infusions (0.2 μl/side, infused at 0.1 μl/min). The injectors were left in place for 1 min after the end of the infusion to allow for diffusion and avoid capillary action. Each mouse received three microinjections, with a 48-h interval between them (**Figure 1A**), in a design that counterbalanced saline and drug treatments. Doses were chosen based on previous studies (Hwa et al., 2013; Albrechet-Souza et al., 2015). Bottles containing ethanol or water were presented to the animals 10 min post-infusion.

### Blood Ethanol Concentration (BEC) Analysis and Histology

After the last test day, mice were given continuous access to ethanol and water for 48 h before being deeply anesthetized with an overdose of ketamine (300 mg/kg) + xylazine (30 mg/kg, i.p.). Blood samples were collected by cardiac puncture, centrifuged at 4°C for 10 min at 3000 rpm and plasma was stored at -80°C for further analysis. Plasma was analyzed for BEC using gas chromatography (Toxilab; Porto Alegre). Next, the animals were perfused with 0.9% saline and 4% paraformaldehyde solution prior to removal of the brains. These procedures occurred during the light phase of the light-dark cycle, between 0900 h and 1200 h. The fixed brains were sliced in 50-μm coronal section using a cryostat. The brain slices were stained with hematoxylin–eosin, and the injector placements were verified by light microscopy, according to the mouse brain atlas (Paxinos and Franklin, 2001). Mice with injector tracks that did not terminate within the BNST were excluded from the analysis (*n* = 2).

### CRF System mRNA Expression in the BNST

Separate cohorts of mice were exposed to the 10-day episodic social defeat protocol as previously described (see Section Social Defeat Stress). Twenty-four hour after the last social defeat, non-stressed controls and stressed mice were euthanized by cervical dislocation (**Figure 1B**). The brains were removed immediately by decapitation and the BNST was bilaterally extracted with a 2-mm-diameter punch tool from a 1 mm tissue slice brain matrix according to Paxinos and Franklin (2001). The tissue punch was then frozen on dry ice and stored at -80°C until used for gene expression analysis.

Total RNA was isolated using QIAzol (Qiagen; Hilden, Germany) and chloroform standard protocols. RNA concentration was measured using Qubit RNA High Sensitivity Assay. Fifty nanograms of RNA from each sample was reverse transcribed using the miScript II RT Kit (Qiagen). The following Quantitect primers (Qiagen) were used: CRF (QT0029389), CRFR1 (QT00106232), CRFR2 (QT00151543), and GAPDH (QT01658692). Each SYBR Green PCR reaction was run in duplicate for each sample and was repeated one time using a Rotor Gene Real-Time PCR machine (Qiagen). The fold change relative expression was calculated using the ΔΔCt method (Livak and Schmittgen, 2001) with the control non-stressed group as a reference. GAPDH ct values were used as endogenous control for mRNA analysis. To verify primer specificities, melting curve analyses were performed.

### Statistical Analysis

Statistical analyses were performed using STATISTICA version 6.0. Descriptive statistics for all measurements are reported as mean ± SEM. Student’s unpaired *t*-tests were used to assess differences in activity in the EPM (% open arms entries, time into the open arms and frequency of closed arms entries), immobility in the TST, BECs and mRNA expression between non-stressed controls and stressed mice.

Body weight, sweet solution/water intake and ethanol/water consumption of non-stressed controls and stressed mice were compared over the sessions of social defeat stress (body weight: day 1-10) or drinking sessions (sweet solution/water intake: BL, SD1, SD10 and 7 days after SD10; ethanol/water consumption: week 1-4) with two-way repeated measures analyses of variance (ANOVAs). To obtain a measure that corrected for individual differences in body weight, grams of ethanol consumed per kilogram of body weight were calculated. Four-week average intakes for individual control mice were compared with 4-week average intakes for individual stressed mice. Two-way repeated measures ANOVAs were also performed, followed by *a priori* driven one-way ANOVA to compare treatments effect (saline, CP376,395 and astressin2B) using each condition (non-stressed or stressed) as a single factor. In case of significance, *post hoc* comparisons were performed using the Newman–Keuls test, a stepwise multiple comparisons procedure based on the Studentized range distribution. Values of *p* < 0.05 were considered statistically significant.

## Results

### Social Defeat Stress Did Not Promote Changes in Body Weight

**FIGURE 2:**
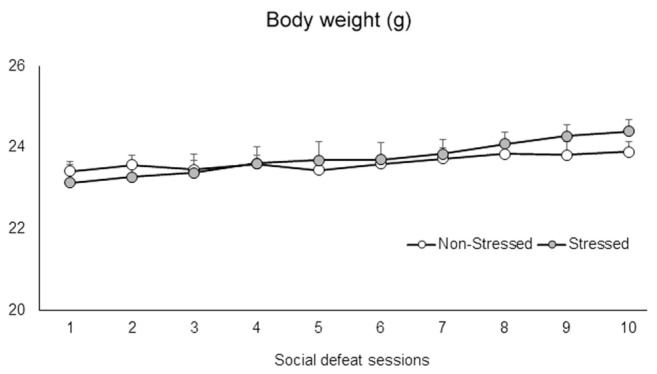
Body weight (g) of non-stressed controls and socially defeated mice. Control non-stressed mice were weighed daily, while stressed mice were weighed and then socially defeated for ten consecutive sessions. Data are mean ± SEM. *n* = 36-37 mice per group.

The defeat phase of the social defeat stress lasted on average 25 s. Mice were not injured by this mild social defeat protocol. A two-way repeated measures ANOVA failed to find significant differences in the body weight of the mice between groups [*F*(1,71) = 0.98, *p* = 0.33], sessions [*F*(9,639) = 0.97, *p* = 0.46], or interaction between factors [*F*(9,639) = 0.97, *p* = 0.46] (**Figure 2**).

### Mice Drank More Sweet Solution after 10 Sessions of Social Defeat Stress

**FIGURE 3:**
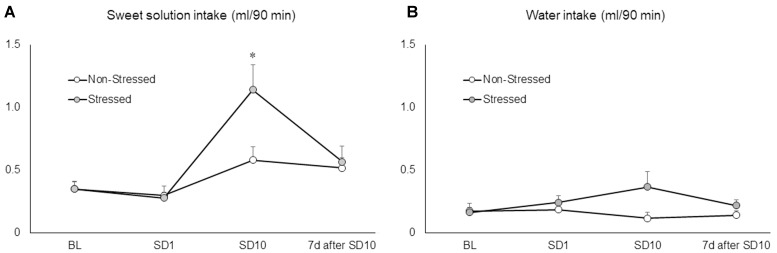
**(A)** Sweet solution (ml/90 min) and **(B)** water intake (ml/90 min) measured in non-stressed controls and socially defeated mice before the beginning of the social defeat stress (baseline conditions, BL), after the first (SD1) and last social defeat session (SD10), and again 7 days after the last confrontation (7 days after SD10). BL corresponds to the average of three 90-min drinking sessions. Data are mean ± SEM. ^∗^versus non-stressed controls and BL. *p* < 0.05, *n* = 12 mice per group.

Ten brief episodes of social defeat stress engendered a significant increase in sweet solution consumption by stressed mice compared to non-stressed controls and baseline conditions as revealed by two-way repeated measures ANOVA followed by Newman–Keuls test [*F*(3,66) = 5.53, *p* = 0.00 on the interaction between conditions and sessions] (**Figure 3A**). The same analysis showed no significant differences in fluid consumption after the first episode of social defeat stress. Moreover, sweet solution intake was measured again 7 days after the last confrontation, at which time there was no longer a significant difference between stressed and non-stressed mice. The two-way repeated measures ANOVA failed to find significant differences on water intake between groups [*F*(1,22) = 2.98, *p* = 0.10], sessions [*F*(3,66) = 0.78, *p* = 0.51], or interaction effects [*F*(3,66) = 2.19, *p* = 0.10] (**Figure 3B**). These results showed that, instead of promoting anhedonia-like symptoms, ten brief episodes of social defeat stress produced an increase in hedonic responses to a palatable solution.

### Stressed Mice Presented Reduced Activity in the Open Arms of the EPM

**FIGURE 4:**
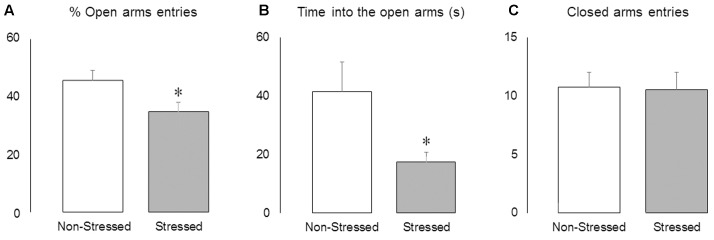
Activity in the elevated plus-maze. **(A)** Percentage of open arms entries, **(B)** time spent into the open arms (s) and **(C)** frequency of closed arms entries in non-stressed controls and socially defeated mice 8 days after the last confrontation. Data are mean ± SEM. ^∗^versus non-stressed controls. *p* < 0.05, *n* = 12 mice per group.

The Student’s *t*-test revealed that brief episodes of social defeat stress promoted anxiety-like responses, decreasing the percentage of open-arm entries [*t* = 2.33, *p* = 0.03] (**Figure 4A**) and time spent into the open arms of the maze [*t* = 2.21, *p* = 0.04] (**Figure 4B**), without changing the frequency of closed arms entries [*t* = 0.13, *p* = 0.90] (**Figure 4C**).

### Social Defeat Stress Did Not Alter Immobility in the TST

**FIGURE 5:**
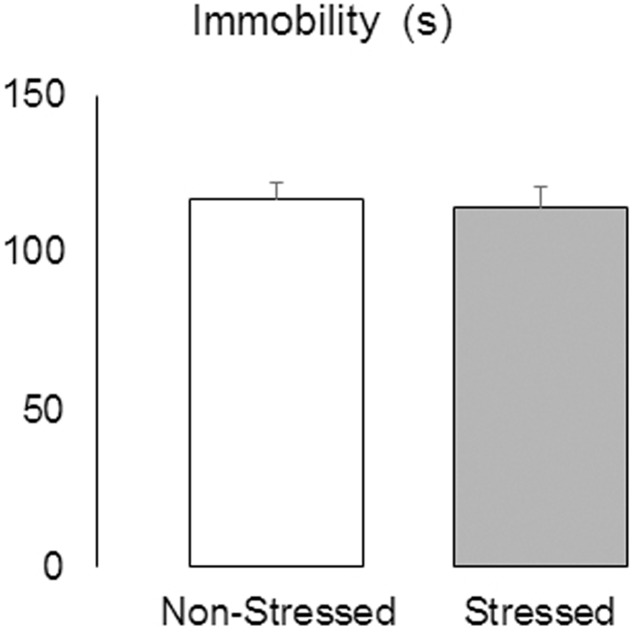
Immobility (s) measured in the tail suspension test in non-stressed controls and socially defeated mice 9 days after the last confrontation. Data are mean ± SEM. *n* = 11-12 mice per group.

The Student’s *t*-test revealed no significant difference between groups in the duration of immobility measured in the TST [*t* = 0.28, *p* = 0.78] (**Figure 5**), suggesting that the resident-intruder protocol used here did not elicit this type of depression-related behavior.

### Social Defeat Stress Promoted an Increase in Voluntary Ethanol Drinking

**FIGURE 6:**
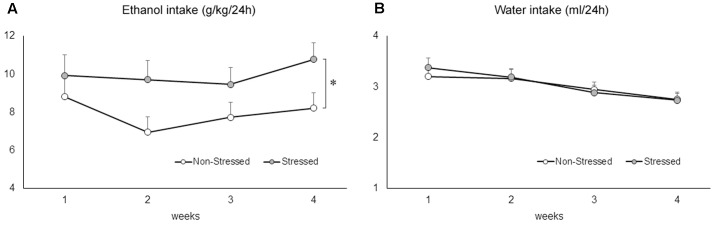
**(A)** Ethanol (g/kg/24 h) and **(B)** water (ml/24 h) consumption in non-stressed controls and socially defeated mice 10 days after the last confrontation. Mice were exposed to continuous access to ethanol (20% weight/volume) and water for 4 weeks. Data are mean ± SEM. ^∗^versus non-stressed controls. *p* < 0.05, *n* = 23-25 mice per group.

The two-way repeated measures ANOVA followed by Newman–Keuls test revealed that stressed mice drank significantly more ethanol compared to non-stressed controls exposed to the two-bottle free-choice paradigm [*F*(1,46) = 5.95, *p* = 0.02] (**Figure 6A**). Ethanol consumption remained significantly elevated in mice with a stress history across the weeks [*F*(3,138) = 3.19, *p* = 0.02]. A two-way repeated measures ANOVA followed by Newman–Keuls test revealed a significant decrease of water intake across the weeks of drinking [*F*(3,138) = 25.63, *p* = 0.00], but failed to find a difference between groups [*F*(1,46) = 0.03, *p* = 0.85], or an interaction effect [*F*(3,138) = 1.09, *p* = 0.36] (**Figure 6B**).

### Stressed Mice Had an Increase in *CRF* mRNA Expression in the BNST

**FIGURE 7:**
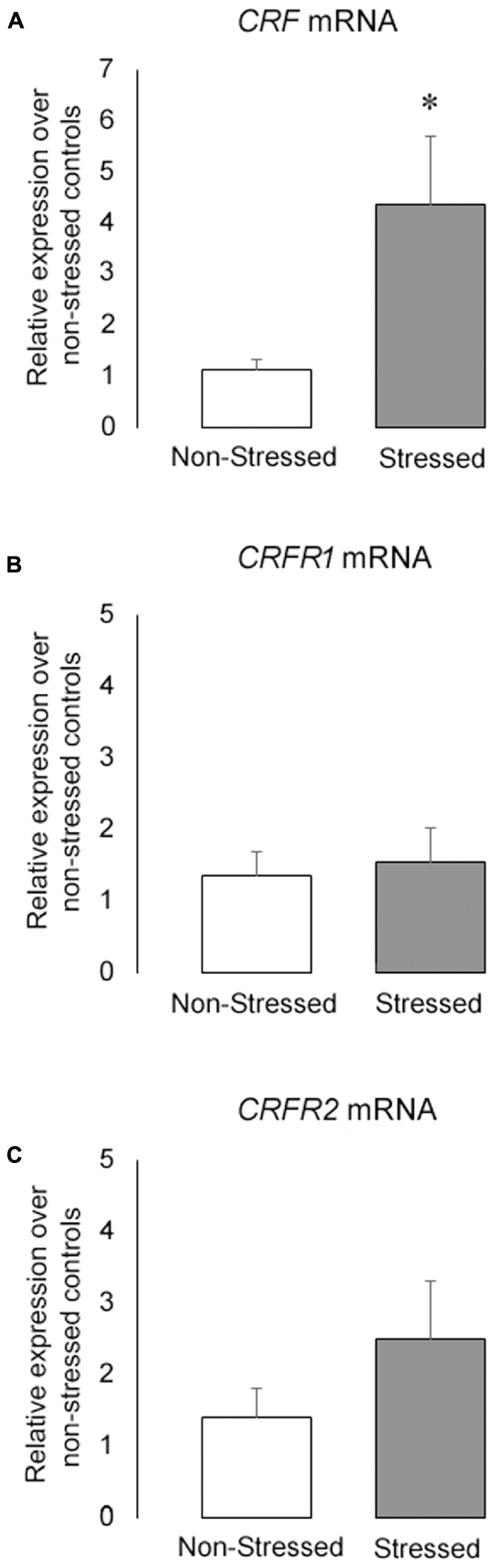
**(A)** *CRF* **(B)** *CRFR1* and **(C)** *CRFR2* mRNA levels in the BNST of non-stressed controls and socially defeated mice measured by qPCR. Gene expression was normalized to GAPDH using the ΔΔCt method and relative to control non-stressed group. Data are mean ± SEM. ^∗^versus non-stressed controls. *p* < 0.05, *n* = 6 mice per group.

The **Figure 7** shows (**Figure 7A**) *CRF*, (**Figure 7B**) *CRFR1* and (**Figure 7C**) *CRFR2* mRNA levels in the BNST in non-stressed controls and stressed mice submitted to ten brief episodes of social defeat stress. Stressed mice presented a significant increase in *CRF* mRNA levels in the BNST compared to controls [*t* = 2.42, *p* = 0.03]. On the other hand, there was no statistical differences in *CRFR1* [*t* = 0.35, *p* = 0.73] and *CRFR2* [*t* = 1.20, *p* = 0.25] mRNA expression between groups.

### Intra-BNST CRF Receptor Antagonists Produced Opposite Effects on Ethanol Drinking

**FIGURE 8:**
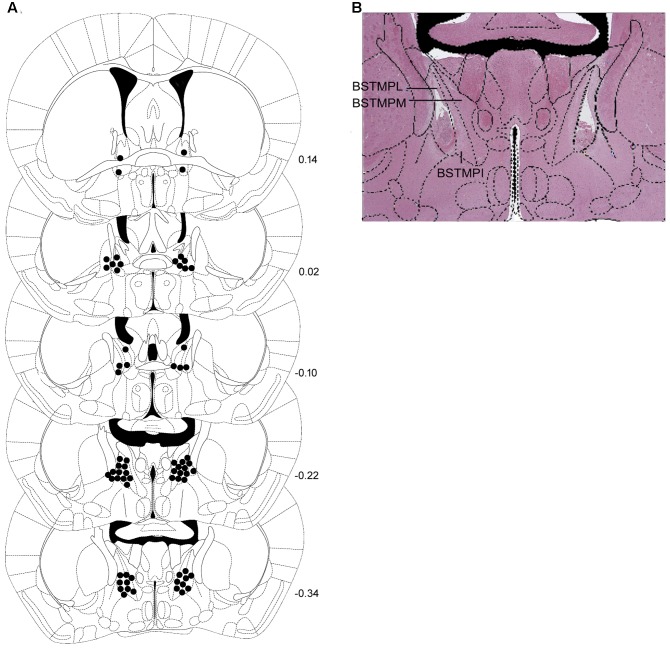
**(A)** Correct placements of intra-BNST bilateral cannulae in non-stressed controls and stressed mice and **(B)** representative photomicrograph after hematoxylin-eosin staining. Each diagram corresponds to a coronal section of the mouse brain according to the bregma (Paxinos and Franklin, 2001). The number of points in the figures is less than the total number of animals because of overlapping injection sites. BSTMPI, bed nucleus of the stria terminalis, medial division, posterointermediate part; BSTMPL, bed nucleus of the stria terminalis, medial division, posterolateral part; BSTMPM, bed nucleus of the stria terminalis, medial division, posteromedial part.

Schematic representations of bilateral injection sites in the BNST, as well as a representative photomicrograph are shown in **Figures 8A,B**.

Two-way ANOVAs revealed significant differences between non-stressed and stressed groups in ethanol intake 2 h [*F*(1,121) = 5.75, *p* = 0.02], 4 h [*F*(1,121) = 3.91, *p* = 0.04] and 24 h [*F*(1,121) = 5.69, *p* = 0.02] post-microinjection. Moreover, there is a significant treatment effect at the 4 h [*F*(4,121) = 3.33, *p* = 0.01] and 24 h [*F*(4,121) = 7.99, *p* = 0.00] time points.

**FIGURE 9:**
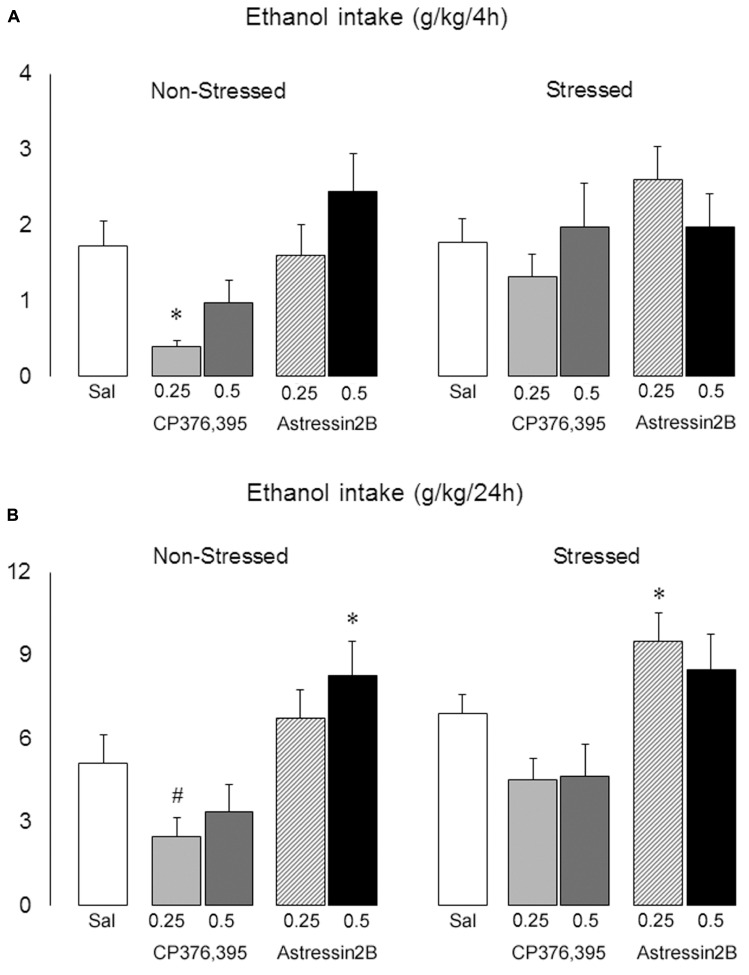
Effects of intra-BNST CRFR1 (CP376,395, CP) or CRFR2 (Astressin2B, A2B) antagonists on ethanol consumption in non-stressed controls and socially defeated mice exposed to continuous access to ethanol/water for 4 weeks. After infusions, mice were given continuous access to ethanol and water for 2 h, **(A)** 4 h and **(B)** 24 h. The graphs are split into non-stressed and stressed groups. Left bars represent non-stressed mice. Right bars represent socially defeated mice. Left, groups from left to right: Sal, non-stressed controls + saline, *n* = 20; 0.25, non-stressed controls + CP 0.25 μg/side, *n* = 11; 0.5, non-stressed controls + CP 0.5 μg/side, *n* = 11; 0.25, non-stressed controls + A2B 0.25 μg/side, *n* = 9; 0.5, non-stressed controls + A2B 0.5 μg/side, *n* = 9. Right, groups from left to right: Sal, stressed mice + saline, *n* = 24; 0.25, stressed mice + CP 0.25 μg/side, *n* = 12; 0.5, stressed mice + CP 0.5 μg/side, *n* = 11; 0.25, stressed mice + A2B 0.25 μg/side, *n* = 12; 0.5, stressed mice + A2B 0.5 μg/side, *n* = 12. Data are mean ± SEM. ^∗^versus Sal group in the same condition (Non-stressed or Stressed, *p* < 0.05); ^#^versus Non-stressed + Sal group (*p* = 0.06).

The one-way ANOVA followed by Newman–Keuls test showed that, in non-stressed mice, intra-BNST injections of CP376,395 at the dose of 0.25 μg decreased ethanol intake at 4 h post-infusion [*F*(4,55) = 4.21, *p* = 0.00] (**Figure 9A**), and produced a trend in reducing ethanol consumption at 24 h post-infusion [*p* = 0.06] (**Figure 9B**). On the other hand, astressin2B at 0.5 μg promoted a significant increase in ethanol intake 24 h post-infusion [*F*(4,55) = 4.02, *p* = 0.01] (**Figure 9B**). In stressed mice, intra-BNST injections of CP376,395 at the doses of 0.25 and 0.5 μg modestly lowered drinking at 24 h post-infusion, although this was not statistically significant [*p* = 0.07 and 0.10, respectively]. Similarly to non-stressed mice, astressin2B at 0.25 μg produced a significant increase in ethanol intake 24 h post-infusion in stressed animals [*F*(4,31) = 4.02, *p* = 0.00] (**Figure 9B**).

**FIGURE 10:**
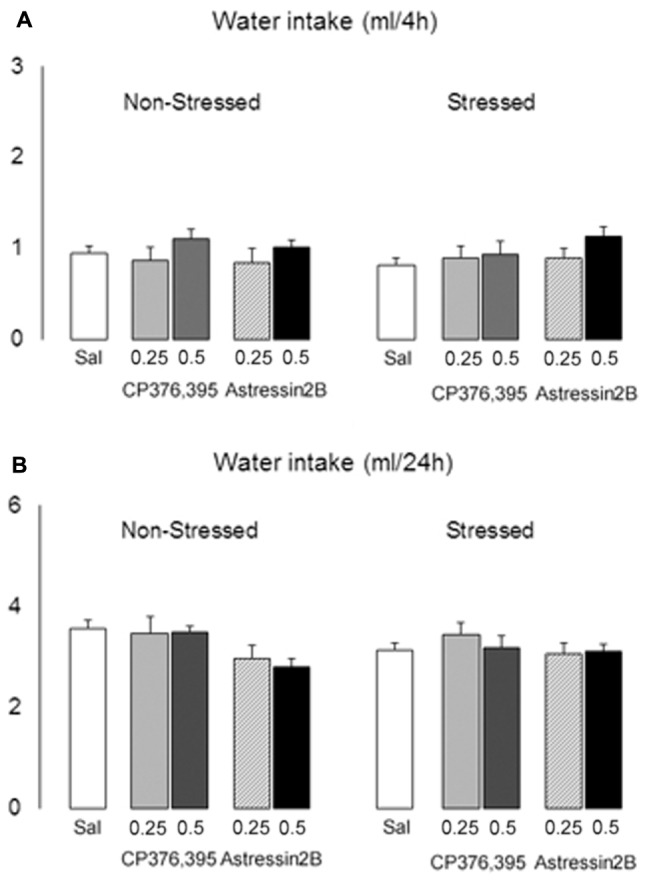
Effects of intra-BNST CRFR1 (CP376,395, CP) or CRFR2 (Astressin2B, A2B) antagonists on water consumption in non-stressed controls and socially defeated mice exposed to continuous access to ethanol/water for 4 weeks. After infusions, mice were given continuous access to ethanol and water for 2 h, **(A)** 4 h and **(B)** 24 h. The graphs are split into non-stressed and stressed groups. Left bars represent non-stressed mice. Right bars represent socially defeated mice. Left, groups from left to right: Sal, non-stressed controls + saline, *n* = 20; 0.25, non-stressed controls + CP 0.25 μg/side, *n* = 11; 0.5, non-stressed controls + CP 0.5 μg/side, *n* = 11; 0.25, non-stressed controls + A2B 0.25 μg/side, *n* = 9; 0.5, non-stressed controls + A2B 0.5 μg/side, *n* = 9. Right, groups from left to right: Sal, stressed mice + saline, *n* = 24; 0.25, stressed mice + CP 0.25 μg/side, *n* = 12; 0.5, stressed mice + CP 0.5 μg/side, *n* = 11; 0.25, stressed mice + A2B 0.25 μg/side, *n* = 12; 0.5, stressed mice + A2B 0.5 μg/side, *n* = 12. Data are mean ± SEM.

Two-way ANOVAs failed to reveal differences between non-stressed and stressed groups in water intake 2 h [*F*(1,121) = 1.71, *p* = 0.19], 4 h [*F*(1,121) = 0.07, *p* = 0.80] and 24 h [*F*(1,121) = 0.23, *p* = 0.60] post-microinjection. Similarly, there is no significant treatment effect at the 2 h [*F*(4,121) = 0.50, *p* = 0.74], 4 h [*F*(4,121) = 1.45, *p* = 0.22] and 24 h [*F*(4,121) = 2.06, *p* = 0.09] time points (**Figures 10A,B**).

**FIGURE 11:**
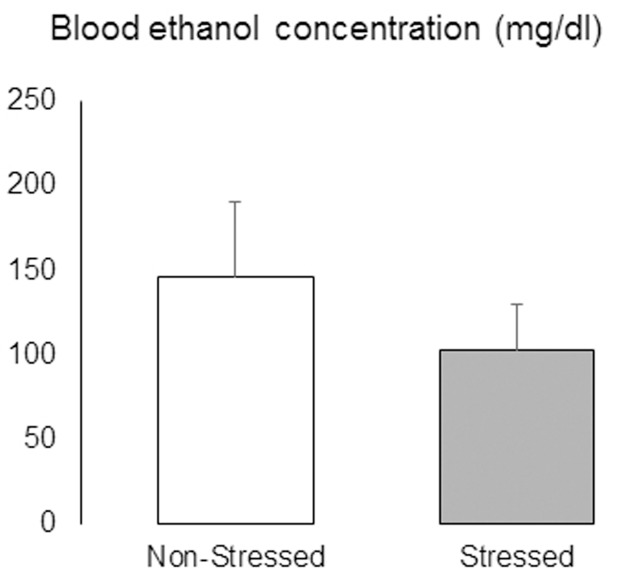
Blood ethanol concentrations (mg/dl) in non-stressed controls and socially defeated mice with a history of continuous access to ethanol. After the last test day, mice were given continuous access to ethanol and water for 48 h before being deeply anesthetized and had blood samples collected by cardiac puncture. Data are mean ± SEM. *n* = 11 mice per group.

After the last test day, mice were given continuous access to ethanol and water for 48 h before being deeply anesthetized and had blood samples collected by cardiac puncture. There was no significant difference in BEC between non-stressed and stressed mice [*t* = 0.83, *p* = 0.41] (**Figure 11**).

## Discussion

Stress has long been hypothesized to be a major factor in the development and maintenance of alcohol abuse (Cloninger, 1987). In the present study, ten brief episodes of social defeat stress engendered anxiety-like behaviors and increased free-choice home cage ethanol drinking in adult C57BL/6 mice. Moreover, previously stressed animals showed higher *CRF* mRNA expression in the BNST compared to non-stressed controls. The antagonism of CRFR1 receptors in the BNST effectively reduced alcohol consumption in non-stressed mice with a history of continuous access to ethanol. We also observed an unexpected increase in alcohol drinking after intra-BNST microinjection with astressin2B in both control and stressed mice.

In order to investigate the effect of stress on behaviors with hedonic motivation we tested the consumption of a palatable sweet solution prepared with 0.1% saccharin and sodium cyclamate. This non-stressful and non-invasive protocol allows repeated tests without compromising the animal’s behavior (Rygula et al., 2005). Classically, the reduction in sweet solution and other palatable food intake has been interpreted as an index of anhedonia, the lack or disruption of the ability to experience pleasure (Willner et al., 1992). Anhedonia is considered one of the core symptoms of major affective disorders according to the DSM-5 (American Psychiatric Association, 2013). However, corroborating our hypothesis, the brief episodes of social defeat stress protocol used here did not reduce the intake of sweet solution. Stressed mice drank more palatable fluid after ten confrontations, probably driven by an anxiety state induced by repeated episodes of social defeat stress. This effect, however, was no longer detectable 7 days after the last social defeat. Decreasing of appetitive activity has been more frequently associated with chronic stress models, which produce some cardinal features of depressive-like symptoms (Willner et al., 1987; Miczek et al., 2011; Shimamoto et al., 2011). Thus, the lack of anhedonia, in association to the absence of changes in body weight and immobility in the TST in stressed mice suggest that the social defeat protocol used in the present study did not induce a depressive-like state in adult C57BL/6 mice.

Ten consecutive days of episodic social defeat stress have been demonstrated to increase plasma corticosterone in outbred mice (Norman et al., 2015). It is noteworthy that certain types of stress and glucocorticoids seem to increase palatable food intake in rodents (Dallman et al., 2003; Pecoraro et al., 2004). Thus, stressful conditions have been proposed to induce or maintain higher incentive salience toward high palatable food as an adaptive coping mechanism acting to reduce the activity in the stress-response network with its attendant anxiety (Dallman et al., 2003; Pecoraro et al., 2004). Interestingly, the CRF-CRFR1 system has been demonstrated to be a key mediator of the excessive eating of palatable food resulting from its intermittent access (Cottone et al., 2009; Iemolo et al., 2013). Thus, in line with the idea that excessive palatable food intake may result as a form of ‘self-medication’ to relieve negative emotional states (Dallman et al., 2005), in the present study stressed mice showed reduced activity in the open arms of the EPM compared to non-stressed controls, indicating the development of a putative anxiety-like state.

Exposure to social defeat stress reliably and persistently increased subsequent alcohol intake compared to non-stressed controls. In contrast to the protocol used by Norman et al. (2015), in the present study mice were exposed to a substantially milder social defeat procedure (direct confrontation: mean = 25 s; 5 bites). An important distinction between these studies is the use of distinct mouse strains; whereas Norman et al. (2015) used outbred CFW mice as both residents and intruders, we used inbred C57BL/6 as intruders and Swiss mice as residents. These results support the idea that biological variables such as sex, age, and genotype may play a significant role in determining the stress effects on behavioral outcomes (Becker et al., 2011). Moreover, although it is generally acknowledged that stressful life events play a prominent role in influencing alcohol drinking, how stress modulates neurobiological systems underlying motivational aspects of alcohol-related behaviors seems to depend on the nature as well as the intensity of the stressor (Pacak and Palkovits, 2001; van Erp et al., 2001; Funk et al., 2005; Miczek et al., 2008; Becker et al., 2011; Norman et al., 2015). Therefore, an important aspect of the present study is that, in contrast with more severe social stress procedures, we identified that five attack bites can be sufficient to consistently escalate alcohol drinking for at least 4 weeks. Moreover, the increase in ethanol intake in stressed mice cannot be explained by a general increase in appetite, because the consumption of sweet solution measured 1 week after the last social defeat was at the same level as that of non-stressed controls.

In this study, socially defeated mice presented increased *CRF* mRNA expression in the BNST, without altering the expression of *CRF* receptors mRNA. In line with these results, the BNST and CRF have been implicated in sustained, but not phasic threat responses (Walker et al., 2009), and a recent study has demonstrated that rats exposed to unpredictable chronic mild stress for 14 consecutive days present increased *CRF* mRNA in the BNST (de Andrade et al., 2017). Taken together, our findings demonstrated that repeated episodes of social defeat stress lead to the development of dysregulated behaviors such as persistent increases of anxiety and excessive ethanol intake, as well as neuroadaptations of the CRF system. Although we have not evaluated directly the role of intra-BNST CRF antagonists on anxiety-like behaviors, we hypothesize that they may be associated with increased CRF expression. In fact, CRF given centrally has been shown to induce anxiogenic behaviors in various animal models, including the EPM (Baldwin et al., 1991). Interestingly, intra-BNST injection of CP376,395 was unable to change the behavioral profile of mice exposed to the EPM without a history of previous stress (Faria et al., 2016).

To explore a mechanistic link between CRF and ethanol drinking, the selective CRFR1 antagonist CP376,395 and the selective CRFR2 antagonist astressin2B were infused into the BNST. CRF signaling via CRFR1 seems to be particularly important in conditions of excessive alcohol taking and seeking, including during early and protracted withdrawal, relapse, as well as during withdrawal-induced anxiety (Hwa et al., 2016; Quadros et al., 2016). In this study, however, CP376,395 effectively reduced alcohol drinking in non-stressed mice relative to saline treatment. Although this was not statistically significant, CP376,395 microinjections also led to a decrease in ethanol intake in stressed mice. By contrast, BNST infusions of astressin2B dose-dependently increased ethanol intake in both non-stressed controls and stressed mice.

While extensive evidence points to a critical role for CRFR1 on ethanol consumption (Lowery-Gionta et al., 2012; Hwa et al., 2013, 2016; Quadros et al., 2016), few studies have investigated the involvement of CRFR2 in the modulation of alcohol drinking. Infusion of Ucn1 (a non-selective agonist at CRFR1/2) into the lateral septum, but not into the dorsal raphé, blunts binge alcohol drinking in mice, presumably due to a preferential action on CRFR2 (Ryabinin et al., 2008). Intraventricular administration of the CRFR2 selective agonist Ucn3 dose-dependently decreased binge drinking, and the administration of the same compound into the central nucleus of the amygdala decreased alcohol self-administration in alcohol-dependent rats (Lowery and Thiele, 2010; Phillips et al., 2015). On the other hand, we have recently demonstrated that intra-VTA astressin2B decreased alcohol consumption in the drinking-in-the-dark paradigm, in which mice were given limited access to 20% ethanol in the dark phase of their circadian cycle, resulting in drinking to intoxication and pharmacologically relevant BEC (Albrechet-Souza et al., 2015). Overall, these results suggest that CRF in the BNST may not specifically underlie exaggerated drinking observed in stressed mice. Moreover, CRFR2 seems to modulate alcohol drinking in a regionally dependent manner. Further studies will help to identify the exact nature of the CRFR2 signaling.

To the best of our knowledge, our data are the first to provide evidence that BNST CRFR1 and CRFR2 have opposing functions in the regulation of continuous ethanol drinking behavior in C57BL/6 mice. These results support a previous report showing that central CRFR1 activation promotes, whereas CRFR2 activation blunts binge-like ethanol drinking in naïve mice (Lowery et al., 2010), and expand the current literature by indicating the BNST as a possible site of action to CRFergic compounds infused centrally. Similarly, in a recent study, intra-VTA antagonism of CRFR1 and activation of CRFR2 using Ucn3 resulted in decreased binge-like ethanol drinking in mice without a history of previous stress (Rinker et al., 2017). Selective inhibition of CRF neurons in the BNST, which projects to the VTA, also reduces binge-like ethanol consumption (Pleil et al., 2015). Thus, both subtypes of CRF receptors seem to be involved in the modulation of alcohol drinking in rodents. While consistent and extensive evidence weighs toward a critical role of CRFR1, increasing findings suggest that a balance between CRFR1 and CRFR2 activation/blockade is important to determine the final behavioral outcome.

## Conclusion

The present work supports previous evidence that social stress is involved in the onset of psychiatric disorders, such as anxiety and alcohol abuse. Moreover, brief episodes of social defeat stress promoted an increase in *CRF* mRNA levels in the BNST. In addition, we report that the blockade of CRFR1 within the BNST reduces voluntary ethanol intake in non-stressed mice, whereas the antagonism of CRFR2 increases alcohol consumption in both socially defeated and non-stressed mice with a history of chronic intake. Given the critical role of the BNST in the reinforcing actions of drugs and the transition to dependence, a clearer understanding of the involvement of the CRF system may provide insights into the onset and maintenance of alcohol-related behaviors and promote the development of new therapeutic strategies.

## Author Contributions

LA-S, KM, RdA, TV, and RG-O contributed to the conception and design of the study. LA-S conducted the behavioral experiments, analyzed the data and wrote the manuscript. TV conducted mRNA analyses and analyzed the data. KM, RdA, and RG-O revised the manuscript. All authors gave final approval of the version to be published.

## Conflict of Interest Statement

The authors declare that the research was conducted in the absence of any commercial or financial relationships that could be construed as a potential conflict of interest.
